# Acute saccharin infusion has no effect on renal glucose handling in normal rats in vivo

**DOI:** 10.14814/phy2.13804

**Published:** 2018-07-16

**Authors:** Grégory Jacquillet, Edward S. Debnam, Robert J. Unwin, Joanne Marks

**Affiliations:** ^1^ Department of Neuroscience, Physiology & Pharmacology University College London London United Kingdom; ^2^ Centre for Nephrology University College London London United Kingdom

**Keywords:** Glucose, glucose tolerance test, in vivo micropuncture, saccharin

## Abstract

Artificial sweeteners are extensively used by the food industry to replace sugar in food and beverages and are widely considered to be a healthy alternative. However, recent data suggest that artificial sweeteners may impact intestinal glucose absorption and that they might lead to glucose intolerance. Moreover, chronic consumption of artificial sweeteners has also been linked to detrimental changes in renal function. Using an in vivo approach, our study aimed to determine if short‐term infusion of the artificial sweetener saccharin can alter renal function and renal glucose absorption. We show that saccharin infusion does not induce any major change in GFR or urine flow rate at either the whole kidney or single nephron level, suggesting that any reported change in renal function with artificial sweeteners must depend on chronic consumption. As expected for a nondiabetic animal, glucose excretion was low; however, saccharin infusion caused a small, but significant, decrease in fractional glucose excretion. In contrast to the whole kidney data, our micropuncture results did not show any significant difference in fractional glucose reabsorption in either the proximal or distal tubules, indicating that saccharin does not influence renal glucose handling in vivo under euglycemic conditions. In keeping with this finding, protein levels of the renal glucose transporters SGLT1 and SGLT2 were also unchanged. In addition, saccharin infusion in rats undergoing a glucose tolerance test failed to induce a robust change in renal glucose excretion or renal glucose transporter expression. In conclusion, our results demonstrate that saccharin does not induce acute physiologically relevant changes in renal function or renal glucose handling.

## Introduction

Diabetic nephropathy is an important complication of diabetes and a major cause of end‐stage renal disease (UKPDS, 1998). Poorly controlled blood glucose levels and hypertension are thought to accelerate diabetes‐induced kidney failure, which now accounts for up to 40% of patients on dialysis (Lassalle et al. 2015). The rates of development of type 2 diabetes mellitus (T2DM) have increased markedly since 1960 and have been attributed, in part, to increased consumption of sugar‐sweetened food or beverages, resulting in the development of obesity and the metabolic syndrome (Zimmet et al. 2001). From these observations, it has been suggested that replacement of sugar in food with artificial sweeteners, also termed non‐nutritive sweeteners (NNS), may be a beneficial option (Duffey et al. 2012; Tate et al. 2012), since they do not provide any calories and can be consumed in large quantities as an alternative to dietary carbohydrate intake.

However, despite the fact that using artificial sweeteners as a sugar substitute reduces energy intake, their beneficial role is controversial, since their effectiveness for weight management or regulation of energy balance is not clear. In humans, comparative studies on the effects of sugar‐ and artificial‐sweetened foodstuffs on hunger and food consumption have shown either no effect (Rogers et al. 1988; Black et al. 1993; Maersk et al. 2012) or a subsequent increase in weight gain and energy intake (Lavin et al. 1997; King et al. 1999) – see Rogers et al. (2016) for a recent and comprehensive review. In rodents, the effect of NNS on weight management or regulation of energy balance is even more questionable. Most animal studies have failed to show that the consumption of artificial sweeteners induces loss of weight (Bailey et al. 1997; Andrejic et al. 2012). Moreover, there are recent reports describing greater weight gain in rats fed with saccharin (or aspartame) that is independent of caloric intake and unrelated to insulin resistance (Feijo Fde et al. 2013; Foletto et al. 2016).

Furthermore, it has been shown that artificial sweeteners contained in diet soda can have an adverse effect on glucose levels and may increase the risk of developing metabolic syndrome and diabetes, due in part to the dysregulation of glucose homeostasis (Nettleton et al. 2009; Imamura et al. 2016). Recent animal studies provide information that supports an active metabolic role of artificial sweeteners in the small intestine. It has been suggested that augmented glucose absorption occurs in response to activation of the taste receptor complex T1R2/3 by NNS (Mace et al. 2007; Margolskee et al. 2007), and that alterations to the gut microbiota caused by NNS consumption may also increase the risk of developing glucose intolerance (Suez et al. 2014).

Concerning the kidney, the results of the few published studies do not provide clear evidence as to whether NSS can affect renal glucose handling. While, the sweet taste receptors, T1R2 and T1R3, have been identified in whole mouse kidney (Rajkumar et al. 2014), they not appear to be present in the tubules per se (Lee et al. 2015), and their precise localization and physiological role have yet to be defined. Intriguingly, it has been shown that acute saccharin infusion increases glucose uptake and SGLT1 protein levels in isolated renal brush border membrane vesicles in vitro (Chichger et al. 2016), and in vivo glucose tolerance tests showed that rats fed saccharin‐sweetened yogurt for 14 days had higher blood glucose levels than animals fed unsweetened yogurt (Swithers et al. 2012). Based on these observations, the aim of this study was to determine whether the artificial sweetener saccharin has any acute effect on renal function and renal glucose reabsorption in normal rats in vivo.

## Materials and Methods

### Ethic approval

All protocols were approved by University College London (Royal Free Campus) Comparative Biology Unit Animal Welfare and Ethical Review Body (AWERB) committee and the procedures were carried out in accordance with the UK Animals (Scientific Procedures) Act, 1986, Amendment Regulations 2012.

### Renal free‐flow micropuncture

Experiments were performed on 30 male Wistar rats purchased from Charles River aged 12–14 weeks (250–300 g). Rats were fed a standard commercial rat chow ad libitum (Diet RM1, SDS Ltd, Witham, UK) and allowed free access to water at all times. After a week of acclimatization, rats were anesthetized with Inactin (120 mg kg^−1^ i.p.; Sigma Ltd., Poole, Dorset, UK) and monitoring of the pedal and corneal reflex was undertaken to ensure that deep anesthesia was achieved before they were surgically prepared for micropuncture as previously described (Vekaria et al. 2006). Throughout each experiment, rats were infused intravenously with a NaCl solution (150 mmol L^−1^) alone (baseline control), or with NaCl solution containing 5 mmol L^−1^ of saccharin, at a rate of 4 mL h^−1^. After a 1 h equilibration period, an i.v. bolus of ^3^H‐inulin (40 *μ*Ci) was given, followed by infusion of ^3^H‐inulin at a rate of 40 *μ*Ci h^−1^ for a further 1 h equilibration period. Micropuncture collections, using sharpened glass micropipettes (tip diameter 8–11 *μ*m) that were filled with Sudan black‐stained oil, were made from middle and late proximal tubules, and early and late distal tubules using methods described previously (Walter and Shirley 1986). Micropuncture collected samples were deposited onto a watch glass under oil for subsequent volume measurement and aliquots taken for analysis. During the experimental period arterial blood and urine were collected from the femoral artery, the bladder and the ureter, respectively. Blood was centrifuged (6000 *g*, 3 min) immediately after collection and plasma was snap frozen. Kidneys were removed at the end of each experiment and snap frozen for subsequent preparation of renal BBM vesicles for use in Western blotting.

### Intravenous glucose tolerance test

Experiments were performed on 12 male Wistar rats purchased from Charles River aged 12–14 weeks (250–300 g). Rats were fed a standard commercial rat chow ad libitum (Diet RM1, SDS Ltd, Witham, UK) and allowed free access to water at all times. To perform these experiments the protocol developed by Nowell and Howland (1966) was adapted to mimic the micropuncture protocol described above. After cannulation of the left jugular vein, right femoral artery and the bladder, a control 0.3 mL sample of blood was withdrawn from the artery and the intravenous infusion of either a NaCl solution (150 mmol L^−1^) alone (control), or with NaCl solution containing 5 mmol L^−1^ of saccharin, at a rate of 4 mL h^−1^ was immediately started. After a 120 min equilibration period, a glucose load (0.2 mL per 100 g BW of a 30% glucose solution) was injected into the left jugular vein. The first test sample was taken 3 min later, and further samples at 10 min intervals for 50 min. Blood was centrifuged (6000 *g*, 3 min) immediately after collection and plasma was snap frozen. Kidneys were removed at the end of each experiment and snap frozen for subsequent preparation of renal BBM vesicles for use in Western blotting.

### Preparation of renal BBM

Brush border membrane vesicles were prepared from kidney cortex, using a double Mg^2+^ chelation protocol, as described previously (Marks et al. 2003). The purity of the BBM preparation was confirmed by six‐ to eight‐fold enrichment of alkaline phosphatase. The protein concentration was determined using a Bradford assay (Bradford 1976).

### Western blotting

Western blotting of the BBM was carried out as previously described (Marks et al. 2003) using rabbit polyclonal antibodies raised against SGLT1 (a kind gift from Professor G. Kellett, University of York, UK) and SGLT2 (a kind gift from Professor H Koepsell, University of Wurzburg, Germany). Mouse monoclonal antibody for *β*‐actin (ab6376, Abcam, Cambridge, UK) was used as a loading control. Blots were visualized with enhanced chemiluminescence on a Fluor‐S MultiImager system (Bio‐Rad, Hemel Hempstead, UK), and the abundance of each protein of interest was calculated relative to *β*‐actin and expressed as a percentage of the control average.

### Analytical procedure

Concentrations of glucose in plasma and urine were determined using a glucose oxidase assay (Huggett and Nixon 1957), and in tubular fluid (TF) by microfluorometry using a nanoflo (WPI, Sarasota, FL). Scintillation counting of ^3^H‐inulin levels in urine, plasma and tubular fluid allows determination of glomerular filtration rate (GFR) and single nephron (SN)GFR. Values were then used to calculate fractional glucose reabsorption at each micropuncture site using standard formulae.

#### Calculations

$$\text{GFR}=\frac{U_{\mathrm{inulin}}}{P_{\mathrm{inulin}}}\times \text{urinary flow rate}\;\text{(mL}\;{\text{min)}}^{-1}$$

$$\text{SNGFR}=\frac{{\text{TF}}_{\mathrm{inulin}}}{P_{\mathrm{inulin}}}\times \text{TF flow rate}\;\text{(nL}\;{\text{min)}}^{-1}$$

$$\begin{array}{cc} & \text{Fractional excretion (FE) of glucose}\\ & \;\;=\frac{U/P_{\mathrm{glucose}}}{U/P_{\mathrm{inulin}}}\times 100\left(\%\right) \end{array}$$

$$\begin{array}{cc} & \text{Fractional reabsorption (FR) of glucose}\\ & \;\;=100-\text{FE}(100\%) \end{array}$$

$$\begin{array}{cc} & \text{Quantity of glucose reabsorbed}\\ & \;\;=P_{\mathrm{glucose}}\times \text{GFR}\times \text{FR}\;\;\left(\mu \text{mol}\;{\text{min}}^{-1}\right) \end{array}$$

### Statistical analysis

Data are presented as mean ± SD. Statistical analysis was performed using either an unpaired Student's *t*‐test, one‐way ANOVA with Bonferroni's post hoc test where relevant, or a two‐way ANOVA with unpaired Student's *t*‐test where relevant. All analyses were performed using GraphPad Prism 5 software, and statistical significance depicted as *P < 0.05, **P < 0.01, ***P < 0.001 or ****P < 0.0001.

## Results

### Renal free‐flow micropuncture

**Figure 1:**
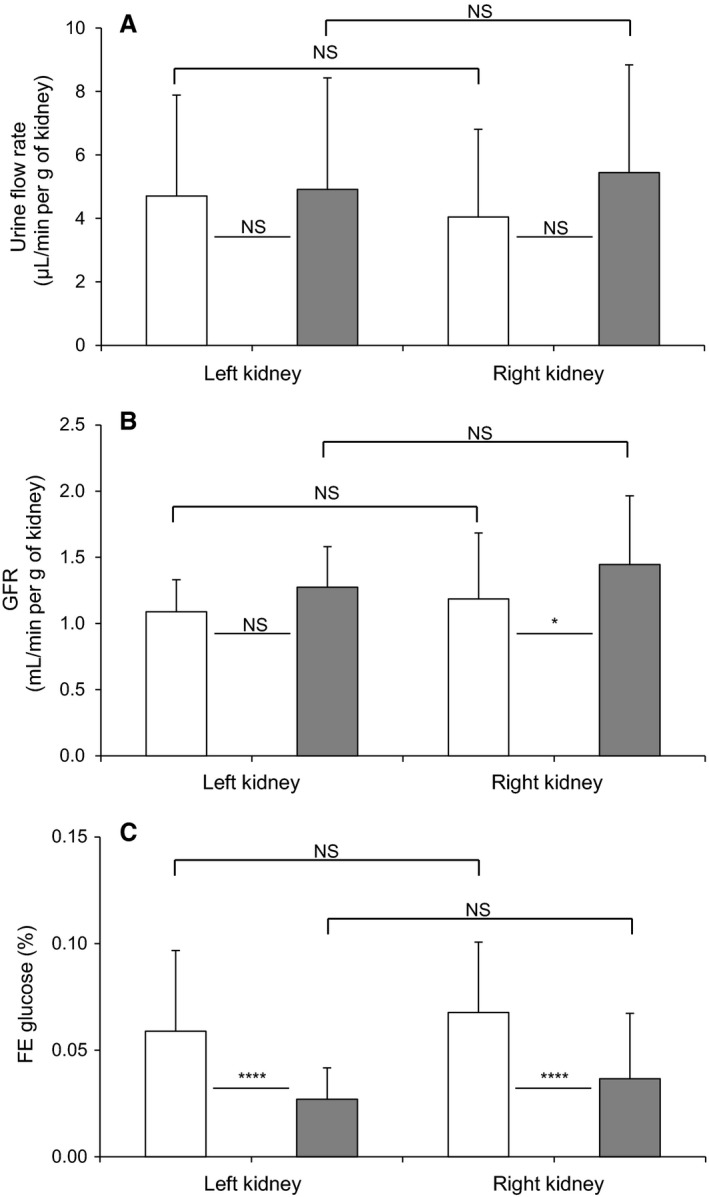
Effect of intravenous saccharin infusion on urinary flow rate (A), glomerular filtration rate (GFR; B), and fractional excretion of glucose (FE; C). Clearance experiments were performed in Wistar rats exposed to intravenous infusion of saline (open bars) or saccharin (filled bars). Results for urinary flow rate and GFR were normalized per gram of kidney. Values are expressed as means ± SD (saline infusion: *n* = 54 and saccharin infusion: *n* = 42), and statistical significance tested using a one‐way ANOVA followed by Bonferroni's post hoc test; NS, not significantly different, *P < 0.05, ****P < 0.0001.

Short‐term saccharin infusion had no effect on plasma glucose concentration (control: 5.0 ± 0.4 vs. saccharin: 5.5 ± 0.4 mmol L^−1^, *n* = 13–17, *P* = 0.32). Saccharin infusion did not affect urine flow rate (Fig. 1A), but there was a small and significant increase in glomerular filtration rate when measured in the right kidney (Fig. 1B). Although as expected for nondiabetic animals, absolute urinary glucose excretion was low, saccharin infusion caused a small but significantly decrease in fractional excretion of glucose (Fig. 1C), suggesting that there may have been some adaptation in renal glucose reabsorption.

**Figure 2:**
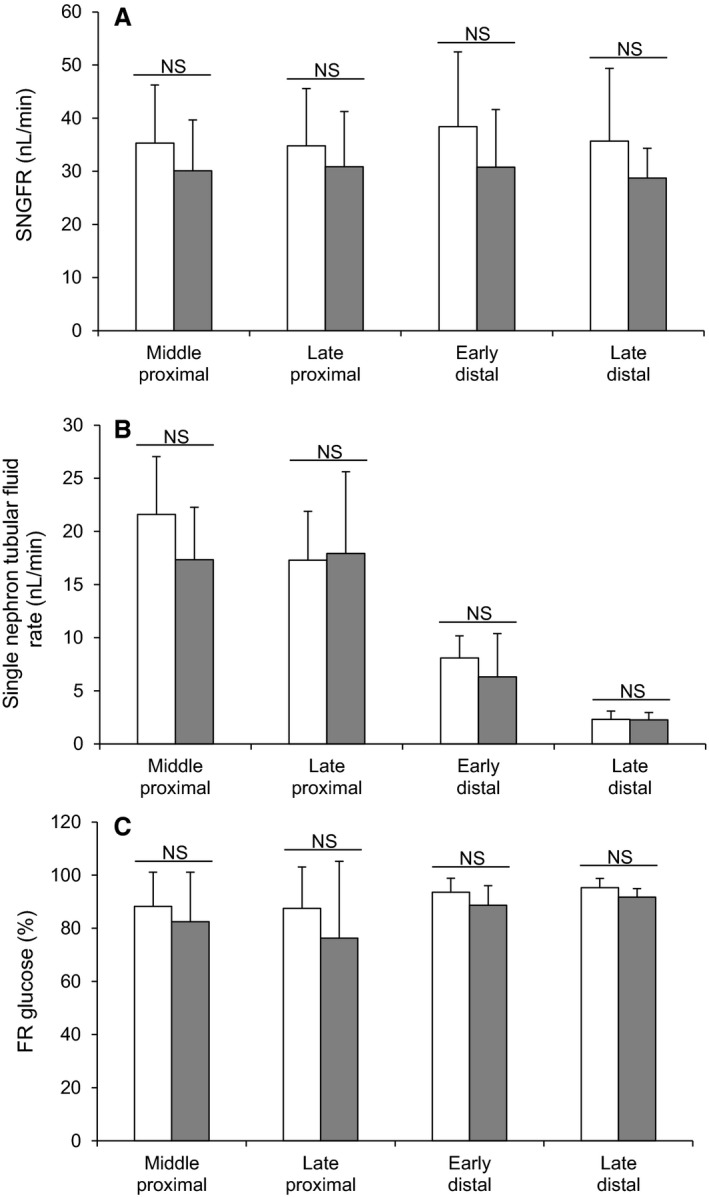
Effect of intravenous saccharin infusion on single nephron glomerular filtration rate (SNGFR; A), single nephron tubular fluid rate (B), and fractional reabsorption of glucose (FR; C). Micropuncture experiments were performed from middle proximal convoluted tubules to late distal tubules in Wistar rats exposed to intravenous infusion of saline (open bars) or saccharin (filled bars). Values are expressed as means ± (saline infusion: middle proximal *n* = 31, late proximal *n* = 27, early distal *n* = 9, late distal *n* = 6 – saccharin infusion: middle proximal *n* = 19, late proximal *n* = 18, early distal *n* = 7, late distal *n* = 4), and statistical significance tested using a one‐way ANOVA followed by Bonferroni's post hoc test; NS, not significantly different compared with control rats.

**Figure 3:**
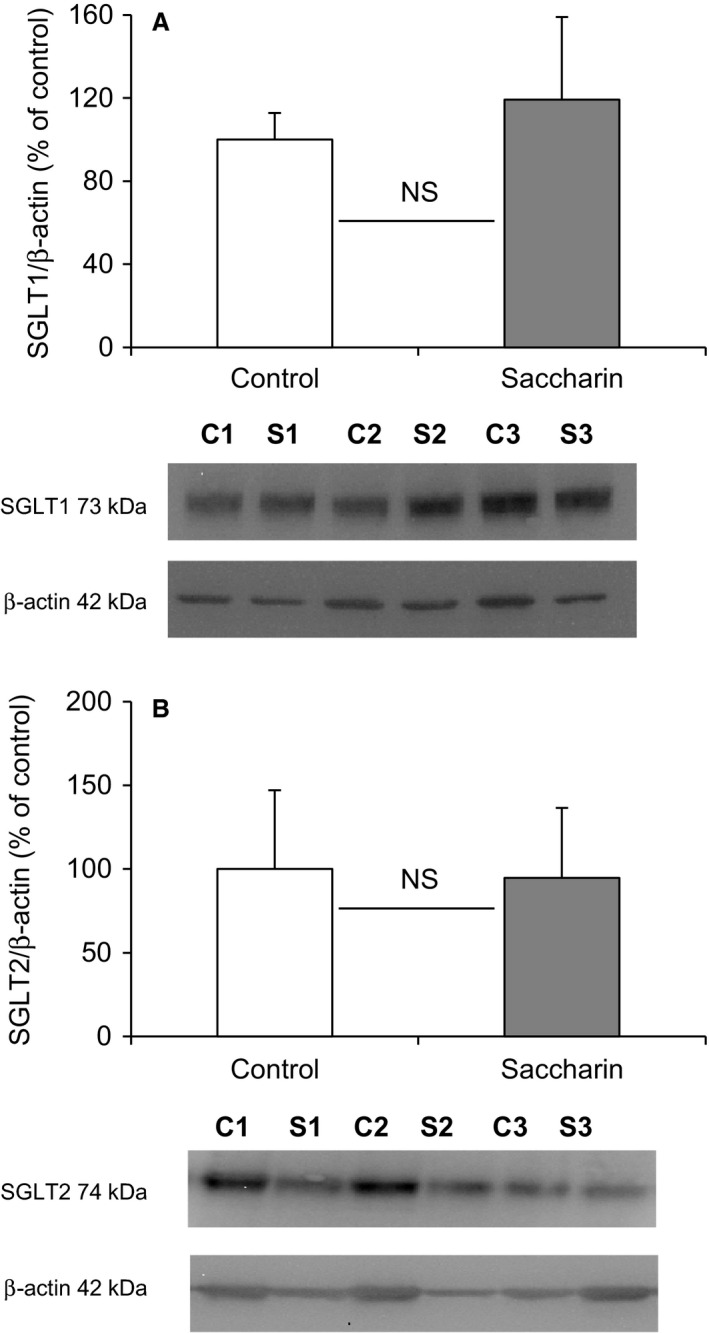
Effect of intravenous saccharin infusion on expression of SGLT1 (A), and SGLT2 (B) at the proximal tubule BBM. Western blotting was performed on renal BBM vesicles prepared from Wistar rats exposed to intravenous infusion of saline (open bars) or saccharin (filled bars). The bar graphs show the density of the protein of interest relative to *β*‐actin and expressed as a percentage of the control average. Results are shown as means ± SD (*n* = 7) and statistical significance tested using an unpaired *t*‐test; NS.

To investigate the potential site of this alteration in renal glucose reabsorption, micropuncture collections were made from middle and late proximal tubules, as well as early and late distal tubules (Fig. 2). For proximal tubules we identified these sites by injecting Sudan black‐stained oil and counting the number of loops visible before the oil droplet disappeared down the Loop of Henle. Mid proximal tubules were classified as 2–7 loops after the collection site and late proximal as zero loops (Table 1). The distal segments were identified after intravenous injection of Lissamine Green (30 *μ*L of a 5% solution). To verify the reliability of our results, SNGFRs were shown to remain stable in the different segments of the nephron (Fig. 2A), and are similar to those published in the literature (Bishop et al. 1979). In addition, we used the decrease in single nephron tubular fluid rate (Fig. 2B) and tubular fluid/plasma [^3^H]‐inulin concentration ratio (TF/Pin) (Table 1), corresponding to the reabsorption of water, as an additional indicator of the punctured nephron site. As expected, single nephron tubular fluid rate and TF/Pin decreased gradually between the early proximal tubule and the late distal tubule (Fig. 2B and Table 1). Infusion of saccharin did not affect nephron function, since SNGFRs and single nephron tubular fluid rates were unchanged between the two groups of animals (Fig. 2A and B). However, in contrast to what was observed in whole kidney, the micropuncture results do not show any significant difference in fractional glucose reabsorption in either the proximal or distal tubules of saccharin‐infused rats compared with control animals (Fig. 2C). In keeping with this finding, Western blotting revealed no significant difference in the levels of SGLT1 and SGLT2 (Fig. 3A and B) in renal BBM vesicles prepared from saccharin infused and control animals.

**Table 1:** Identification of the different micropuncture collection sites using the number of surface loops beyond the collection site and tubular fluid/plasma [3H]inulin concentration ratio (TF/Pin)

		Number of loops		TF/Pin	
		Control	Saccharin	Control	Saccharin
Middle	Average	2–5	2–7	1.7	1.8
Proximal	SD			0.5	0.6
	*n*	31	19	31	19
Late	Average	0	0	2.1	1.8
Proximal	SD			0.7	0.4
	*n*	27	18	27	18
Early	Average	n/a	n/a	4.7	5.6
Distal	SD			1.2	1.8
	*n*	9	7	9	7
Late	Average	n/a	n/a	16.7	14.5
Distal	SD			8.2	8.3
	*n*	6	4	6	4

Micropuncture experiments were performed from middle proximal convoluted tubules to late distal tubules in Wistar rats exposed to intravenous infusion of saline (control) or saccharin. Values are expressed as means ± SD, and statistical significance tested using an unpaired *t*‐test.

### Glucose tolerance test

**Figure 4:**
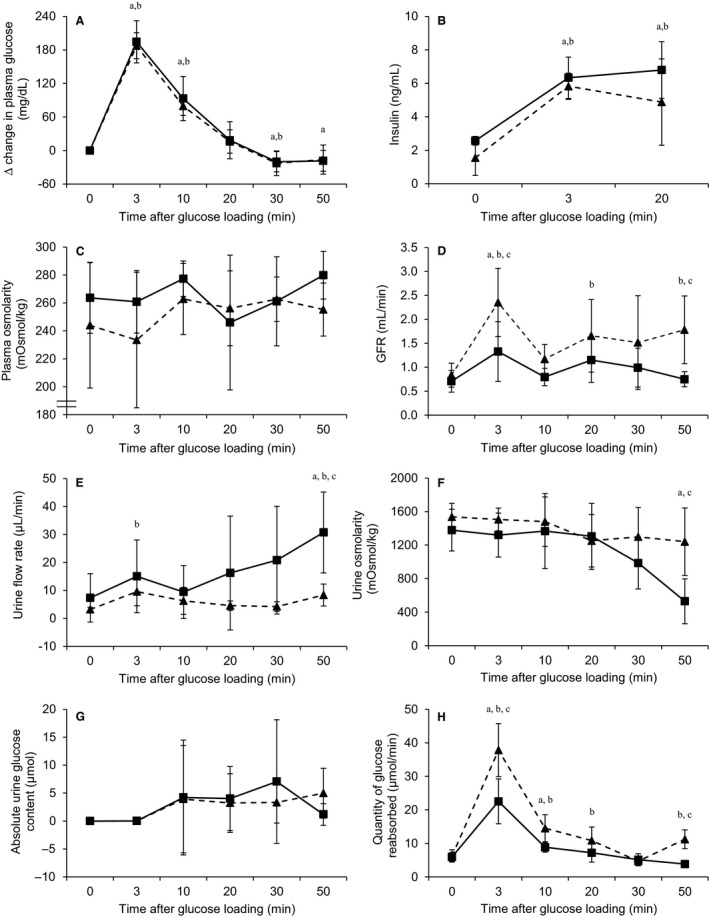
Effect of intravenous saccharin infusion on ∆ change in plasma glucose (A), plasma insulin (B), plasma osmolarity (C), GFR (D), urine flow rate (E), urine osmolarity (F), absolute urine glucose content (G), and quantity of glucose reabsorbed (H) during a glucose tolerance test. Glucose tolerance test was performed in Wistar rats exposed to intravenous infusion of saline (solid line, ■) or saccharin (dash line, ▴). Values are expressed as means ± (saline infusion: *n* = 6 and saccharin infusion: *n* = 6) and statistical significance tested using a two‐way ANOVA followed by *t*‐ test to compare between control and experimental groups over time; “a” indicates a significant difference (*P* < 0.05) from period 0 in control group; “b” indicates a significant difference (*P* < 0.05) from time 0 in saccharin group; “c” indicates a significant difference (*P* < 0.05) between control and saccharin groups.

To establish whether the saccharin‐induced decrease in fractional excretion of glucose by the whole kidney could be amplified by an elevated filtered glucose load, we performed glucose tolerance tests on animals in the presence or absence of saccharin infusion. After the 2 h equilibration period prior to administration of glucose, the fractional excretion of glucose was significantly decreased in saccharin‐treated animals compared with control, 0.13 ± 0.06 and 0.04 ± 0.03%, respectively, *n* = 6, *P* = 0.016. As expected, a rise in plasma glucose concentration was observed following an i.v. bolus of 30% glucose solution, with a maximum level 3 min after the injection (Fig. 4A). This change in plasma glucose concentration was associated with an increase in the secretion of insulin by the pancreas (Fig. 4B), but did not change plasma osmolality (Fig. 4C). Furthermore, saccharin infusion had no effect on any of these parameters (Fig. 4A–C).

**Figure 5:**
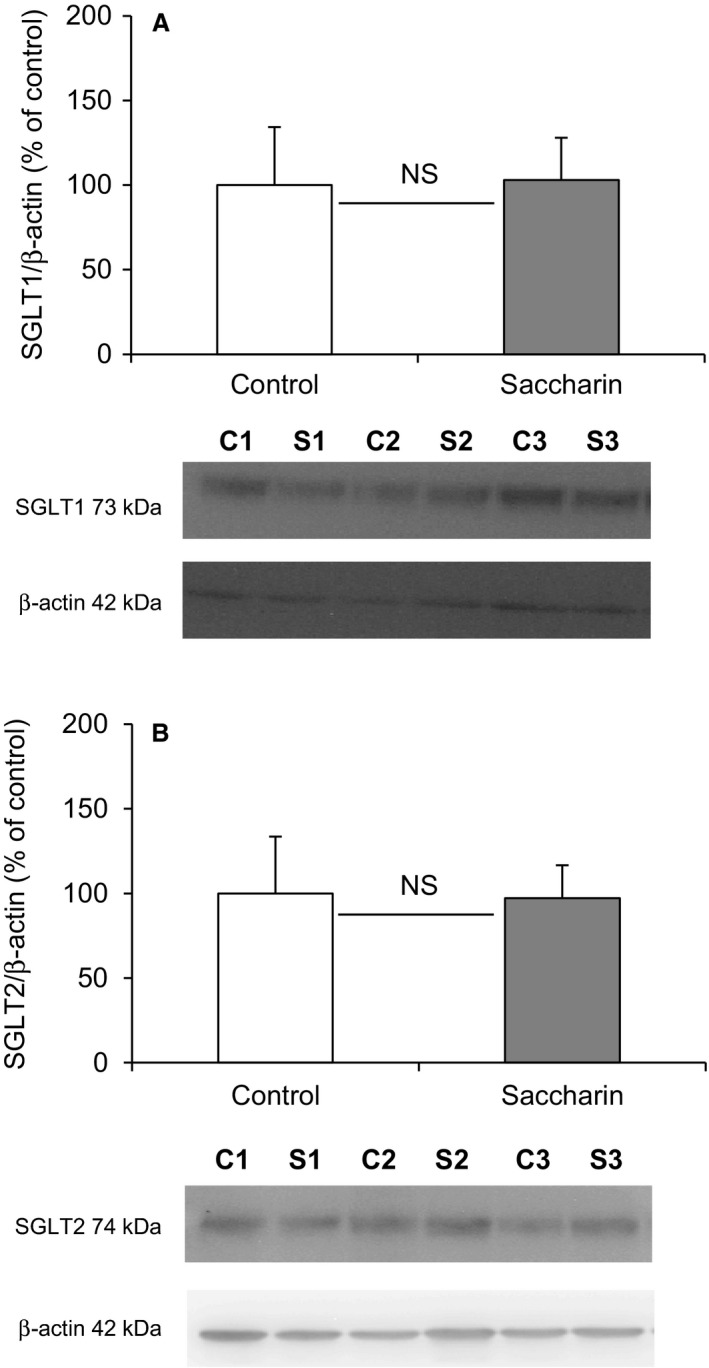
Effect of intravenous saccharin infusion on expression of SGLT1 (A), and SGLT2 (B) at the proximal tubule BBM during a glucose tolerance test. Western blotting was performed on renal BBM vesicles prepared from Wistar rats having undergone a glucose tolerance test and exposed to intravenous infusion of saline (open bars) or saccharin (filled bars). The bar graphs show the density of the protein of interest relative to *β*‐actin and expressed as a percentage of the control average. Results are shown as means ± SD (*n* = 6) and statistical significance tested using an unpaired *t*‐test; NS, not significantly different.

In contrast, in saccharin‐infused animals, GFR increased after glucose loading and remained higher than baseline (*t* = 0); in control animals the bolus of glucose did not produce a sustained effect on GFR (Fig. 4D). GFR was significantly higher in the saccharin group compared with controls at 3 and 50 min after glucose loading (Fig. 4D). Urine flow rate was also affected by saccharin infusion: in controls urine flow rate increased after 10 min from 9.45 ± 9.49 *μ*L min^−1^ to a peak of 30.76 ± 14.46 *μ*L min^−1^ at 50 min (Fig. 4E). The rise in urinary flow rate in control animals would be an expected osmotic diuretic effect, which appears to be blunted after saccharin infusion, since both flow rate and urine osmolality remained unchanged (Fig. 4E and F, respectively). In control animals their urinary glucose concentration increased after glucose loading and reached a maximum 30 min after the injection, before returning to baseline after 50 min (Fig. 4G). Overall, although saccharin infusion did not significantly affect absolute urine glucose content, it is interesting to note that at 50 min urinary glucose levels were still elevated compared with the control (Fig. 4G). Similarly, the quantities of glucose reabsorbed by the kidney increased after glucose loading and were significantly higher in the saccharin‐infused animals compared with control rats (Fig. 4H). At 50 min, similar to what was observed with urinary glucose concentration, the quantity of glucose reabsorbed in the saccharin group was still elevated compared with the control (Fig. 4H). However, Western blotting revealed that saccharin infusion had no significant effect on the levels of SGLT1 and SGLT2 in BBM vesicles following a glucose load (Fig. 5A and B).

## Discussion

Currently, our understanding of the impact of artificial sweetener consumption on renal function is limited. It is known that saccharin is freely filtered by the glomerulus and is cleared by the kidney (Ball et al. 1977), and that this is achieved not only by filtration, but also by active secretion along the nephron (Goldstein et al. 1978; Bekersky et al. 1980; Bourgoignie et al. 1980). Interestingly, it has been reported recently that chronic ingestion of saccharin is associated with an increase in serum urea and creatinine levels, indicating that this NNS may affect renal function (Abdelaziz and Ashour Ael 2011; Amin et al. 2016). In addition, studies have suggested that saccharin consumption induces polyuria in both rodents (Anderson et al. 1988) and humans (Seibert et al. 2011), although the latter is linked to extreme levels of NNS consumption and seems to be reversible. Therefore, the first aim of our study was to establish the impact of short‐term infusion of saccharin on renal function using a renal clearance and free‐flow micropuncture approach. We showed that short‐term saccharin infusion did not induce any major change in GFR or urine flow rate at either the whole kidney or single nephron level, highlighting that any alteration in renal function is probably associated with only chronic consumption of NNS.

The second aim of the study was to investigate the impact of saccharin on renal glucose reabsorption and glucose homeostasis. The rationale for this was based on the fact that the cellular mechanisms for renal and intestinal glucose handling display striking similarities (Marks et al. 2003), and previous studies have reported that NNS consumption affects intestinal glucose absorption, and that sweeteners may be nutritionally active (Mace et al. 2007). It was shown in rats that trafficking of GLUT2 to the enterocyte apical membrane was enhanced by different artificial sweeteners, including saccharin, and that this occurred via sweet taste receptor sensing (Mace et al. 2007). Artificial sweeteners were also reported to increase GLUT2, but not SGLT1‐mediated transport in human and rat enterocyte‐like cell lines (Zheng and Sarr 2013). In contrast, while Margolskee et al. demonstrated that artificial sweeteners enhance intestinal glucose absorptive capacity in mice, this is associated with increased SGLT1, and not GLUT2, protein levels (Margolskee et al. 2007). However, it should be noted that more recent studies in rats (Chaudhry et al. 2013) and healthy human subjects (Ma et al. 2010) have been unable to confirm a role for artificial sweeteners in the augmentation of intestinal glucose absorption. Contradictory effects of NNS on glucose homeostasis have also been described: in vivo glucose tolerance tests revealed that rats fed saccharin‐sweetened yogurt for 14 days had higher blood glucose levels than animals fed unsweetened yogurt (Swithers et al. 2012); in contrast, Amin et al. have reported recently that oral administration of low and high doses of saccharin (10 and 500 mg kg^−1^ body wt.) to rats for 30 days induces a decrease in blood glucose (Amin et al. 2016). Interestingly, neither of these effects has been replicated in humans (Ma et al. 2010; Bryant and McLaughlin 2016). Finally, NNS consumption has been linked to changes in the balance and diversity of the gut microbiota, which has been proposed to enhance the risk of developing glucose intolerance in both mice and humans (Suez et al. 2014; Nettleton et al. 2016).

To our knowledge the impact of NNS on renal glucose transport has not been investigated in any detail. Our initial and earlier studies suggested that glucose uptake was increased in BBM vesicles prepared from saccharin‐infused Sprague–Dawley rats compared with saline‐infused animals, and that this correlated with an increase in SGLT1 protein levels (Chichger et al. 2016). However, the effect of saccharin on renal glucose handling in vivo and the impact of any associated glycemic excursion remain unknown. In this study, the fractional excretion of glucose in normal euglycemic saccharin‐treated Wistar rats was significantly lower, suggesting an increase in glucose reabsorption. While this is in keeping with our previous in vitro findings, it should be noted that fractional reabsorption was already at 99% in both groups, bringing into question the physiological relevance of this minor augmentation. In this context, if saccharin can induce a robust and significant physiological response, we would have expected renal micropuncture to confirm this, as well as the site and transport mechanism(s) responsible for any alterations in renal glucose handling. However, in contrast to what was observed in whole kidney, our micropuncture results did not show any difference in fractional glucose reabsorption at either proximal or distal tubular sites. The discrepancy between these data can probably be explained by the fact that the 0.03% difference in fractional glucose excretion by the whole kidney in response to saccharin is a sum of the function of the 1‐1.5 million nephrons present in each kidney, and that the small change, and heterogeneity, that may occur in each nephron to produce a summed effect at the whole kidney level, is not detectable by micropuncture, again suggesting that saccharin does not significantly influence renal glucose handling in vivo.

In keeping with this interpretation, but in contrast to our previous finding, was the result that saccharin given systemically and acutely did not alter protein levels of the apical renal glucose transporters SGLT1 or SGLT2. However, the discrepancy between the two studies is difficult to explain. We believed that our preliminary observations were real, and it was for this reason that this follow‐up study was undertaken. We have used the same BBM vesicle preparation and Western blotting methods, and the same antibodies in each study. While it could be argued that the use of BBM vesicle uptake studies may amplify the impact of saccharin on renal glucose transport to explain the differences between the in vitro and in vivo effects, it does not provide an explanation for the difference in SGLT1 protein levels between the two studies. Possible explanations for the difference between the two studies are the use of 1 mmol L^−1^ versus 5 mmol L^−1^ saccharin, and the use of Wister versus Sprague–Dawley rats. The dose of our original study (1 mmol L^−1^) was chosen based on studies performed by Mace and colleagues investigating the impact of artificial sweeteners on intestinal glucose transport (Mace et al. 2007). They demonstrated that luminal perfusion of 20 mmol L^−1^ glucose plus 1 mmol L^−1^ of the artificial sweeteners sucralose, saccharin or acesulfame potassium induced the same change in intestinal glucose absorption as the presence of 75 mmol L^−1^ glucose, and suggested that the response occurred via activation of sweet taste receptors. It is well established that these receptors are sensitive to sugars at high concentrations (>100 mmol L^−1^), but that the intense sweetness of an artificial sweetener can elicit a response from the receptor at concentrations between 1 and 5 mmol L^−1^ (Nelson et al. 2001; Li et al. 2002). Given our infusion protocol would result in significant dilution of the original starting concentration (as opposed to the intestinal luminal perfusion approach employed by (Mace et al. 2007)), we opted to use a concentration of 5 mmol L^−1^ in this study to maximize any potential response. In addition, we chose Wistar rats for the follow‐up study, because we had planned to test the effect of saccharin in type 2 diabetic, Goto–Kakizaki rats, making Wistar rats the relevant control; however, in light of our present findings, we no longer think it justified to investigate the effect of saccharin in this diabetic model.

Given the potential impact of glycemic excursion on renal glucose reabsorption, we also sought to access whether the effect of saccharin on renal glucose handling would be amplified in response to a glucose tolerance test. To overcome the influence of intestinal glucose absorption and the subsequent stimulation of gut derived incretin hormones during an oral glucose tolerance test, we employed a method using rapid intravenous administration of glucose (Ross and Tonks 1938). Using this approach in both humans and rodents, a rapid increase in blood glucose level is observed, followed by a return to baseline level within 1 h, depending on the dose injected (Ikkos and Luft 1957; Nowell and Howland 1966). In this study we observed the expected elevation in blood glucose levels, with a return to the initial concentration ~30 min after injection; importantly, saccharin infusion did not alter this response. We also demonstrated that short‐term saccharin treatment did not alter the degree of insulin secretion in response to elevated circulating glucose concentration; a finding in keeping with longer term studies using saccharin‐sweetened foods (Swithers et al. 2012). As with the studies in euglycemic rats, saccharin infusion in rats undergoing a glucose tolerance test failed to induce a robust change in renal glucose excretion or renal glucose transporter expression, demonstrating that saccharin does not influence renal glucose handling in vivo, even under hyperglycemic conditions. Although we found that saccharin treatment was associated with a divergent effect of the glucose load on urinary flow rate and urine osmolality, and potentially an elevated GFR, it is not clear if this is an artifact of our methodology, and if not, what the likely physiological consequences might be.

In conclusion, although numerous studies have suggested that chronic saccharin ingestion can affect renal function, we failed to find evidence that short‐term saccharin infusion has any significant effect in vivo. In addition, while there are reports that NNS consumption can impact glucose homeostasis and intestinal glucose absorption, we were unable to demonstrate an effect of acute saccharin infusion on renal glucose handling.

## Conflict of Interest

None declared.

## References

[phy213804-bib-0001] Abdelaziz, I. , and R. Ashour Ael . 2011 Effect of saccharin on albino rats' blood indices and the therapeutic action of vitamins C and E. Hum. Exp. Toxicol. 30:129–137. 20382728 10.1177/0960327110368695

[phy213804-bib-0002] Amin, K. A. , H. M. Al‐muzafar , and A. H. Abd Elsttar . 2016 Effect of sweetener and flavoring agent on oxidative indices, liver and kidney function levels in rats. Indian J. Exp. Biol. 54:56–63. 26891553

[phy213804-bib-0003] Anderson, R. L. , F. R. Lefever , and J. K. Maurer . 1988 The effect of various saccharin forms on gastro‐intestinal tract, urine and bladder of male rats. Food Chem. Toxicol. 26:665–669. 3198034 10.1016/0278-6915(88)90065-8

[phy213804-bib-0004] Andrejic, B. M. , V. M. Mijatovic , I. N. Samojlik , O. J. Horvat , J. D. Calasan , and M. A. Dolai . 2012 The influence of chronic intake of saccharin on rat hepatic and pancreatic function and morphology: gender differences. Bosn J. Basic Med. Sci. 13:94–99. 10.17305/bjbms.2013.2372 PMC4333936 23725505

[phy213804-bib-0005] Bailey, C. J. , C. Day , J. M. Knapper , S. L. Turner , and P. R. Flatt . 1997 Antihyperglycaemic effect of saccharin in diabetic ob/ob mice. Br. J. Pharmacol. 120:74–78. 9117102 10.1038/sj.bjp.0700871 PMC1564347

[phy213804-bib-0006] Ball, L. M. , A. G. Renwick , and R. T. Williams . 1977 The fate of [14C]saccharin in man, rat and rabbit and of 2‐sulphamoyl[14C]benzoic acid in the rat. Xenobiotica 7:189–203. 868078 10.3109/00498257709035778

[phy213804-bib-0007] Bekersky, I. , W. J. Poynor , and W. A. Colburn . 1980 Pharmacokinetics of saccharin in the rat. Renal clearance in vivo and in the isolated perfused kidney. Drug Metab. Dispos. 8:64–67. 6103789

[phy213804-bib-0008] Bishop, J. H. , R. Green , and S. Thomas . 1979 Free‐flow reabsorption of glucose, sodium, osmoles and water in rat proximal convoluted tubule. J. Physiol. 288:331–351. 469722 PMC1281429

[phy213804-bib-0009] Black, R. M. , L. A. Leiter , and G. H. Anderson . 1993 Consuming aspartame with and without taste: differential effects on appetite and food intake of young adult males. Physiol. Behav. 53:459–466. 8451310 10.1016/0031-9384(93)90139-7

[phy213804-bib-0010] Bourgoignie, J. J. , K. H. Hwang , J. P. Pennell , and N. S. Bricker . 1980 Renal excretion of 2,3‐dihydro‐3‐oxobenzisosulfonazole (saccharin). Am. J. Physiol. 238:F10–F15. 6986795 10.1152/ajprenal.1980.238.1.F10

[phy213804-bib-0011] Bradford, M. M. 1976 A rapid and sensitive method for the quantitation of microgram quantities of protein utilizing the principle of protein‐dye binding. Anal. Biochem. 72:248–254. 942051 10.1016/0003-2697(76)90527-3

[phy213804-bib-0012] Bryant, C. , and J. McLaughlin . 2016 Low calorie sweeteners: evidence remains lacking for effects on human gut function. Physiol. Behav. 164:482–485. 27133729 10.1016/j.physbeh.2016.04.026

[phy213804-bib-0013] Chaudhry, R. M. , A. Garg , M. M. Abdelfatah , J. A. Duenes , and M. G. Sarr . 2013 Lack of functionally active sweet taste receptors in the jejunum in vivo in the rat. J. Surg. Res. 183:606–611. 23531453 10.1016/j.jss.2013.02.031 PMC3713171

[phy213804-bib-0014] Chichger, H. , M. E. Cleasby , S. K. Srai , R. J. Unwin , E. S. Debnam , and J. Marks . 2016 Experimental type II diabetes and related models of impaired glucose metabolism differentially regulate glucose transporters at the proximal tubule brush border membrane. Exp. Physiol. 101:731–742. 27164183 10.1113/EP085670

[phy213804-bib-0015] Duffey, K. J. , L. M. Steffen , L. Van Horn , D. R. Jr Jacobs , and B. M. Popkin . 2012 Dietary patterns matter: diet beverages and cardiometabolic risks in the longitudinal Coronary Artery Risk Development in Young Adults (CARDIA) Study. Am. J. Clin. Nutr. 95:909–915. 22378729 10.3945/ajcn.111.026682 PMC3302365

[phy213804-bib-0016] Feijo Fde, M. , C. R. Ballard , K. C. Foletto , B. A. Batista , A. M. Neves , M. F. Ribeiro , et al. 2013 Saccharin and aspartame, compared with sucrose, induce greater weight gain in adult Wistar rats, at similar total caloric intake levels. Appetite 60:203–207. 23088901 10.1016/j.appet.2012.10.009

[phy213804-bib-0017] Foletto, K. C. , B. A. Melo Batista , A. M. Neves , Feijo F. de Matos , C. R. Ballard , M. F. Marques Ribeiro , et al. 2016 Sweet taste of saccharin induces weight gain without increasing caloric intake, not related to insulin‐resistance in Wistar rats. Appetite 96:604–610. 26555482 10.1016/j.appet.2015.11.003

[phy213804-bib-0018] Goldstein, R. S. , J. B. Hook , and J. T. Bond . 1978 Renal tubular transport of saccharin. J. Pharmacol. Exp. Ther. 204:690–695. 147338

[phy213804-bib-0019] Huggett, A. S. , and D. A. Nixon . 1957 Use of glucose oxidase, peroxidase, and O‐dianisidine in determination of blood and urinary glucose. Lancet 273:368–370. 13464070 10.1016/s0140-6736(57)92595-3

[phy213804-bib-0020] Ikkos, D. , and R. Luft . 1957 On the intravenous glucose tolerance test. Acta Endocrinol. (Copenh) 25:312–334. 13443758 10.1530/acta.0.0250312

[phy213804-bib-0021] Imamura, F. , L. O'Connor , Z. Ye , J. Mursu , Y. Hayashino , S. N. Bhupathiraju , et al. 2016 Consumption of sugar sweetened beverages, artificially sweetened beverages, and fruit juice and incidence of type 2 diabetes: systematic review, meta‐analysis, and estimation of population attributable fraction. Br. J. Sports Med. 50:496–504. 27044603 10.1136/bjsports-2016-h3576rep PMC4853528

[phy213804-bib-0022] King, N. A. , K. Appleton , P. J. Rogers , and J. E. Blundell . 1999 Effects of sweetness and energy in drinks on food intake following exercise. Physiol. Behav. 66:375–379. 10336168 10.1016/s0031-9384(98)00280-7

[phy213804-bib-0023] Lassalle, M. , C. Ayav , L. Frimat , C. Jacquelinet , and C. Couchoud . 2015 The essential of 2012 results from the French Renal Epidemiology and Information Network (REIN) ESRD registry. Nephrol. Ther. 11:78–87. 25457107 10.1016/j.nephro.2014.08.002

[phy213804-bib-0024] Lavin, J. H. , S. J. French , and N. W. Read . 1997 The effect of sucrose‐ and aspartame‐sweetened drinks on energy intake, hunger and food choice of female, moderately restrained eaters. Int. J. Obes. Relat. Metab. Disord. 21:37–42. 9023599 10.1038/sj.ijo.0800360

[phy213804-bib-0025] Lee, J. W. , C. L. Chou , and M. A. Knepper . 2015 Deep Sequencing in Microdissected Renal Tubules Identifies Nephron Segment‐Specific Transcriptomes. J. Am. Soc. Nephrol. 26:2669–2677. 25817355 10.1681/ASN.2014111067 PMC4625681

[phy213804-bib-0026] Li, X. , L. Staszewski , H. Xu , K. Durick , M. Zoller , and E. Adler . 2002 Human receptors for sweet and umami taste. Proc. Natl Acad. Sci. USA 99:4692–4696. 11917125 10.1073/pnas.072090199 PMC123709

[phy213804-bib-0027] Ma, J. , J. Chang , H. L. Checklin , R. L. Young , K. L. Jones , M. Horowitz , et al. 2010 Effect of the artificial sweetener, sucralose, on small intestinal glucose absorption in healthy human subjects. Br. J. Nutr. 104:803–806. 20420761 10.1017/S0007114510001327

[phy213804-bib-0028] Mace, O. J. , J. Affleck , N. Patel , and G. L. Kellett . 2007 Sweet taste receptors in rat small intestine stimulate glucose absorption through apical GLUT2. J. Physiol. 582:379–392. 17495045 10.1113/jphysiol.2007.130906 PMC2075289

[phy213804-bib-0029] Maersk, M. , A. Belza , J. J. Holst , M. Fenger‐Gron , S. B. Pedersen , A. Astrup , et al. 2012 Satiety scores and satiety hormone response after sucrose‐sweetened soft drink compared with isocaloric semi‐skimmed milk and with non‐caloric soft drink: a controlled trial. Eur. J. Clin. Nutr. 66:523–529. 22252107 10.1038/ejcn.2011.223

[phy213804-bib-0030] Margolskee, R. F. , J. Dyer , Z. Kokrashvili , K. S. Salmon , E. Ilegems , K. Daly , et al. 2007 T1R3 and gustducin in gut sense sugars to regulate expression of Na+‐glucose cotransporter 1. Proc. Natl Acad. Sci. USA 104:15075–15080. 17724332 10.1073/pnas.0706678104 PMC1986615

[phy213804-bib-0031] Marks, J. , N. J. Carvou , E. S. Debnam , S. K. Srai , and R. J. Unwin . 2003 Diabetes increases facilitative glucose uptake and GLUT2 expression at the rat proximal tubule brush border membrane. J. Physiol. 553:137–145. 12963802 10.1113/jphysiol.2003.046268 PMC2343472

[phy213804-bib-0032] Nelson, G. , M. A. Hoon , J. Chandrashekar , Y. Zhang , N. J. Ryba , and C. S. Zuker . 2001 Mammalian sweet taste receptors. Cell 106:381–390. 11509186 10.1016/s0092-8674(01)00451-2

[phy213804-bib-0033] Nettleton, J. A. , P. L. Lutsey , Y. Wang , J. A. Lima , E. D. Michos , and D. R. Jr Jacobs . 2009 Diet soda intake and risk of incident metabolic syndrome and type 2 diabetes in the Multi‐Ethnic Study of Atherosclerosis (MESA). Diabetes Care 32:688–694. 19151203 10.2337/dc08-1799 PMC2660468

[phy213804-bib-0034] Nettleton, J. E. , R. A. Reimer , and J. Shearer . 2016 Reshaping the gut microbiota: impact of low calorie sweeteners and the link to insulin resistance? Physiol. Behav. 164:488–493. 27090230 10.1016/j.physbeh.2016.04.029

[phy213804-bib-0035] Nowell, N. W. , and R. J. Howland . 1966 The control of blood sugar in the laboratory rat and golden hamster. I. Intravenous glucose tolerance and insulin sensitivity tests. Acta Endocrinol. (Copenh) 52:149–153. 5329332 10.1530/acta.0.0520149

[phy213804-bib-0036] Rajkumar, P. , W. H. Aisenberg , O. W. Acres , R. J. Protzko , and J. L. Pluznick . 2014 Identification and characterization of novel renal sensory receptors. PLoS ONE 9:e111053. 25340336 10.1371/journal.pone.0111053 PMC4207771

[phy213804-bib-0037] Rogers, P. J. , J. A. Carlyle , A. J. Hill , and J. E. Blundell . 1988 Uncoupling sweet taste and calories: comparison of the effects of glucose and three intense sweeteners on hunger and food intake. Physiol. Behav. 43:547–552. 3200909 10.1016/0031-9384(88)90207-7

[phy213804-bib-0038] Rogers, P. J. , P. S. Hogenkamp , C. de Graaf , S. Higgs , A. Lluch , A. R. Ness , et al. 2016 Does low‐energy sweetener consumption affect energy intake and body weight? A systematic review, including meta‐analyses, of the evidence from human and animal studies. Int. J. Obes.s (Lond) 40:381–394. 10.1038/ijo.2015.177 PMC4786736 26365102

[phy213804-bib-0039] Ross, C. W. , and E. L. Tonks . 1938 The determination of glucose tolerance. Arch. Dis. Child. 13:289–309. 21032119 10.1136/adc.13.76.289 PMC1975589

[phy213804-bib-0040] Seibert, F. S. , B. Riesselmann , and T. H. Westhoff . 2011 A sweet cause of polyuria. Am. J. Kidney Dis. 57:355–356. 21251550 10.1053/j.ajkd.2010.11.016

[phy213804-bib-0041] Suez, J. , T. Korem , D. Zeevi , G. Zilberman‐Schapira , C. A. Thaiss , O. Maza , et al. 2014 Artificial sweeteners induce glucose intolerance by altering the gut microbiota. Nature 514:181–186. 25231862 10.1038/nature13793

[phy213804-bib-0042] Swithers, S. E. , A. F. Laboy , K. Clark , S. Cooper , and T. L. Davidson . 2012 Experience with the high‐intensity sweetener saccharin impairs glucose homeostasis and GLP‐1 release in rats. Behav. Brain Res. 233:1–14. 22561130 10.1016/j.bbr.2012.04.024 PMC3378816

[phy213804-bib-0043] Tate, D. F. , G. Turner‐McGrievy , E. Lyons , J. Stevens , K. Erickson , K. Polzien , et al. 2012 Replacing caloric beverages with water or diet beverages for weight loss in adults: main results of the Choose Healthy Options Consciously Everyday (CHOICE) randomized clinical trial. Am. J. Clin. Nutr. 95:555–563. 22301929 10.3945/ajcn.111.026278 PMC3632875

[phy213804-bib-0044] UKPDS . 1998 Intensive blood‐glucose control with sulphonylureas or insulin compared with conventional treatment and risk of complications in patients with type 2 diabetes (UKPDS 33). UK Prospective Diabetes Study (UKPDS) Group. Lancet 352:837–853. 9742976

[phy213804-bib-0045] Vekaria, R. M. , R. J. Unwin , and D. G. Shirley . 2006 Intraluminal ATP concentrations in rat renal tubules. J. Am. Soc. Nephrol. 17:1841–1847. 16790512 10.1681/ASN.2005111171

[phy213804-bib-0046] Walter, S. J. , and D. G. Shirley . 1986 The effect of chronic hydrochlorothiazide administration on renal function in the rat. Clin. Sci. (Lond.) 70:379–387. 3698514 10.1042/cs0700379

[phy213804-bib-0047] Zheng, Y. , and M. G. Sarr . 2013 Effect of the artificial sweetener, acesulfame potassium, a sweet taste receptor agonist, on glucose uptake in small intestinal cell lines. J. Gastrointest. Surg. 17:153–158.discussion p 158. 22948835 10.1007/s11605-012-1998-z PMC3516624

[phy213804-bib-0048] Zimmet, P. , K. G. Alberti , and J. Shaw . 2001 Global and societal implications of the diabetes epidemic. Nature 414:782–787. 11742409 10.1038/414782a

